# Differential Plasma Expression Profiles of Long Non-Coding RNAs Reveal Potential Biomarkers for Systemic Lupus Erythematosus

**DOI:** 10.3390/biom9060206

**Published:** 2019-05-28

**Authors:** Guo-Cui Wu, Yan Hu, Shi-Yang Guan, Dong-Qing Ye, Hai-Feng Pan

**Affiliations:** 1 School of Nursing, Anhui Medical University, 15 Feicui Road, Hefei 230601, China; gcwu82@126.com; 2 Department of Epidemiology and Biostatistics, School of Public Health, Anhui Medical University, 81 Meishan Road, Hefei 230032, China; huyan1104@126.com (Y.H.); gsy92@foxmail.com (S.-Y.G.); 3 Anhui Province Key Laboratory of Major Autoimmune Diseases, 81 Meishan Road, Hefei 230032, China

**Keywords:** biomarker, diagnosis, long non-coding RNA, systemic lupus erythematosus

## Abstract

Identify long non-coding RNAs (lncRNAs) that might serve as biomarkers for systemic lupus erythematosus (SLE) and explore the biological functions of the identified lncRNAs. In the screening phase, we examined the lncRNA expression profile of plasma samples from 24 patients with SLE and 12 healthy controls (HCs) using lncRNA microarray with pooled samples. The candidate lncRNAs were verified in individual samples by quantitative real-time (qRT)-PCR. In the independent validation stage, the identified lncRNAs were evaluated in 240 patients with SLE and 120 HCs. The identified lncRNAs were assessed further in an external validation stage including patients with rheumatoid arthritis (RA) and primary Sjögren’s syndrome (pSS). In addition, we constructed correlated expression networks including coding–non-coding co-expression and competing endogenous RNAs (ceRNAs). Plasma levels of linc0597, lnc0640, and lnc5150 were elevated in SLE patients compared with those of HCs, whereas levels of GAS5 and lnc7074 were decreased. Five lncRNAs were identified as potential SLE biomarkers with an area under the receiver operating characteristic curve (AUC) ranging from 0.604 to 0.833 in the independent validation phase. This panel of five lncRNAs had high diagnostic accuracy for SLE (AUC = 0.966) and distinguished SLE from RA and pSS (AUC = 0.683 and 0.910, respectively). Co-expression analysis showed that GAS5, lnc0640, and lnc5150 may participate in the SLE pathogenesis through the MAPK pathway. The ceRNA network indicated that GAS5, lnc0640, lnc3643, lnc6655, and lnc7074 bind competitively with microRNAs regulating the expression of target genes. Aberrant expression and related pathways suggest the important role of lncRNAs in SLE pathogenesis. In addition, the panel of five lncRNAs (GAS5, lnc7074, linc0597, lnc0640, and lnc5150) in plasma could be used as SLE biomarkers.

## 1. Introduction

Systemic lupus erythematosus (SLE) is a multisystem autoimmune disease of variable severity; its etiology is multifactorial, mainly involving genetic, epigenetic, and environmental factors [1]. It is a heterogeneous disease and no single clinical characteristic or laboratory test is diagnostic. Lupus nephritis (LN), as one of the most severe manifestations of SLE, affects up to 70% of SLE patients and has significant impact on the outcome of SLE patients [2]. The discovery of an effective and reliable tool for better diagnosis of SLE is essential for disease monitoring and prognostication. 

At present, although the exact pathogenesis of SLE remains unclear, it is known that its etiology is multifactorial mainly involving genetic, epigenetic, and environmental factors [1,3]. Recently, several studies have placed a new emphasis on the role of long non-coding RNAs (lncRNAs) with a length longer than 200 nucleotides and poor protein coding potential [4,5,6]. It has become apparent that lncRNAs participate in regulating gene expression by versatile interactions with DNA, RNA, or proteins [7,8]. It has been reported that lncRNAs play important regulatory roles in Toll-like receptor signaling, regulating not only the innate immune response but also immune cell development and adaptive immunity [9,10]. Increasing evidence suggests that lncRNAs play a critical role in the pathogenesis of autoimmune diseases, such as SLE, rheumatoid arthritis (RA), Sjögren’s syndrome (SS), etc. [11,12,13,14,15]. 

The lncRNA growth arrest-specific 5 (GAS5), which is necessary for normal growth arrest, apoptosis, and cell cycle function in T-cell lines and non-transformed lymphocytes [16], has been associated with an increased risk for developing SLE in a mouse model [17]. In addition, GAS5 has been shown to be involved in the development of human SLE [18]. Two other lncRNAs, linc0949 and linc0597, were decreased in peripheral blood mononuclear cells (PBMCs) from SLE patients. Moreover, linc0949 could act as a biomarker for SLE [11]. Nuclear-enriched abundant transcript 1 (NEAT1), as an early lipopolysaccharide response lncRNA, regulates the innate immune response through Toll-like receptor signaling [19,20]. NEAT1 expression was increased in PBMCs of SLE patients and positively correlated with disease activity. Moreover, NEAT1 affected the expression of inflammatory chemokines and cytokines through the activation of the late MAPK signaling pathway [21].

lncRNAs are stable in human plasma and can serve as biomarkers for multiple diseases, such as cancer, cardiovascular diseases, among others [22,23,24,25]. Our previous study showed that a set of plasma lncRNAs may serve as non-invasive biomarkers for SLE [26]. To understand further the potential of plasma lncRNAs as biomarkers for SLE, we screened lncRNAs in pooled plasma samples in the present study from patients with SLE and healthy controls (HCs) using an lncRNA microarray followed by extensive independent validation.

## 2. Materials and Methods

### 2.1. Design and Study Population

The subjects included 264 patients with SLE, 12 HCs, 30 patients with rheumatoid arthritis (RA), and 31 patients with primary Sjögren’s syndrome (pSS), who were recruited from April 2015 to April 2016. The inclusion and exclusion criteria were as described previously [26]. The disease severity was quantified according to the Systemic Lupus Erythematosus Disease Activity Index 2000 (SLEDAI-2K) [27]. Disease activity was quantified using the SLEDAI-2K score. Active SLE was defined as a SLEDAI-2K score >10, whereas those patients with SLEDAI-2K ≤10 were classed as relatively inactive [11,28].

A four-phase case-control study design was applied. Phase 1 – screening: plasma extracted from 24 new-onset SLE patients (including 12 SLE patients with no LN and 12 LN patients) and from 12 age- and sex-matched HCs was applied to a human lncRNA microarray. Phase 2 – preliminary validation: lncRNAs selected from the screening phase were verified in individual samples by a quantitative real-time (qRT)-PCR assay. Phase 3 – independent validation: the remaining 240 patients with SLE and 120 age- and sex-matched HCs were randomly classified as a training set (160 SLE patients vs. 80 HCs) and a testing set (80 SLE patients vs. 40 HCs). The HCs were recruited from the physical examination center of the Second Affiliated Hospital of Anhui Medical University. Phase 4 – external validation: we examined the levels of candidate lncRNAs considered to be potential candidates for SLE biomarkers in 30 patients with RA and 31 patients with pSS to identify the specificity. The study procedure was approved by the Ethics Committee of Anhui Medical University (approval number: 20150115). 

### 2.2. Total RNA Isolation and qRT-PCR 

Total RNA isolation and an lncRNA qRT-PCR assay were conducted as described previously [26]. Total RNA were reverse-transcribed into cDNA with a PrimeScript RT reagent kit (Takara Bio Inc., Shiga, Japan), and the quantitative real-time PCR (qPCR) was performed using an SYBR Premix Ex Taq™ II (Takara Bio Inc.). The relative plasma lncRNA expression level was normalized to the GAPDH expression [29,30,31]. The primers used in qRT-PCR of the lncRNAs are listed in Table S1, Supplementary Materials. The relative expression of lncRNAs was calculated using the 2^-ΔΔCt^ method normalized to endogenous control, with ΔCt = Ct_target_ − Ct_reference_, − ΔΔCt = − (sample ΔCt – control ΔCt) [32]. 

### 2.3. Microarray Analysis of lncRNAs and Data Analysis

In the screening phase, total RNA was isolated from the plasma samples of 12 SLE patients with LN, 12 SLE patients without LN, and 12 HCs. Four RNA samples were mixed together as a pooled sample for microarray testing. Accordingly, three pooled RNA samples from each group were used in microarray screening.

Arraystar Human LncRNA Microarray V3.0 (Arraystar Inc. Rockville, MD, USA) (including 30,586 lncRNAs and 26,109 coding transcripts) was used for the global profiling of human lncRNAs and protein-coding transcripts. Sample labeling and array hybridization were carried out using the Agilent One-Color Microarray-Based Gene Expression Analysis (Santa Clara, CA, USA) protocol with minor modifications. Agilent Feature Extraction software (version 11.0.1.1, Santa Clara, CA, USA) was applied to analyze the acquired array images. Quantile normalization and subsequent data processing were conducted using the GeneSpring GX version 12.1 software package (Agilent Technologies, Santa Clara, CA, USA). The raw microarray data were submitted to the Gene Expression Omnibus public database (accession number: GSE102547).

The threshold for identifying upregulated and downregulated genes was a *p*-value ≤0.05 and a fold change ≥2.0. Hierarchical clustering was conducted to display the distinguishable lncRNA and mRNA expression patterns among samples. Kyoto Encyclopedia of Genes and Genomes (KEGG) analysis was performed to describe the roles of the differentially expressed mRNAs.

### 2.4. Co-Expression Network Analysis

Co-expression analysis was conducted by calculating Pearson’s correlation coefficient (PCC) between the normalized intensity of the differentially expressed mRNAs and lncRNA biomarkers. The co-expression network of lncRNAs and mRNAs was drawn using Cytoscape software version 2.8.1 (Cytoscape Consortium, San Diego, CA, USA), with PCC ≥0.935.

### 2.5. Construction of a Competing Endogenous RNA Network

The construction of a competing endogenous RNA (ceRNA) network included three steps: (i) screening of microRNAs (miRNAs) relevant to SLE using a combination of miRNAs identified in the literature and differentially expressed miRNAs found in our previous study; (ii) the putative interactions of lncRNA–miRNA and miRNA–mRNA were predicted by miRanda version 3.3a [33]; and (iii) on the basis of the predicted miRNA–mRNA and miRNA–lncRNA regulatory pairs, a ceRNA network was generated in which the lncRNAs and mRNAs interacted via shared miRNAs.

### 2.6. Statistical Analysis

Normally distributed data are presented as the mean ± standard deviation. Data with a skewed distribution are expressed as the median (interquartile range). Categorical data are described using frequency or percentage. A Student’s *t*-test was used to compare the means of normally distributed data between groups. A chi-squared test was used to determine the difference in categorical data between groups. One-way analysis of variance (ANOVA) was applied to quantitative data with a normal distribution for comparisons among multiple groups (≥3). Logistic regression was used to calculate probabilities, odds ratios, and corresponding 95% confidence intervals (CIs). Receiver operating characteristic (ROC) curves were constructed and the area under the curve (AUC) was used to evaluate the value of plasma lncRNAs as biomarkers for SLE. A stepwise logistic regression model was used to select diagnostic lncRNA markers based on the data of the training set or combined training-and-testing set. 

All statistical analyses were conducted using SPSS 10.01 (SPSS Inc., Chicago, IL, USA). Scatter diagrams were generated through GraphPad Prism 5.01 (GraphPad Software, Inc., San Diego, CA, USA). ROC curve analysis was conducted by using MedCalc 11.4.2.0 (MedCalc Software, Mariakerke, Belgium). *p*-values ≤0.05 were considered statistically significant. 

## 3. Results

### 3.1. Basic Characteristics of the Study Subjects 

In the microarray screening and preliminary validation phases, the demographic and baseline clinical data of the study subjects were summarized as described previously [26]. The characteristics of the SLE patients and HCs in the independent validation phase are shown in Table 1. The basic features of the disease controls (RA and pSS patients) are summarized in Table 2.

### 3.2. lncRNA and mRNA Expression Profiles in the Plasma of SLE Patients

**Figure 1:**
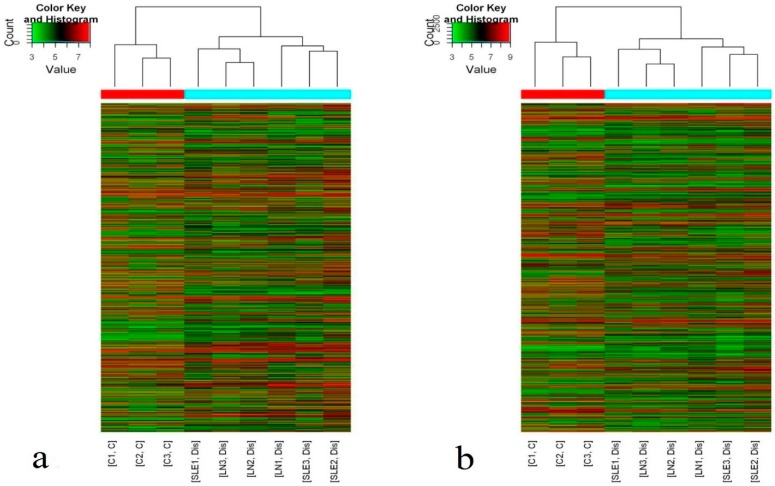
Differential expression of long non-coding RNAs (lncRNAs) and mRNAs between systemic lupus erythematosus (SLE) patients and control subjects. Hierarchical clustering analysis of (**a**) lncRNAs and (**b**) mRNAs that were differentially expressed in plasma of SLE patients (SLE1-3: SLE no LN; LN1-3: lupus nephritis) compared with control samples (C1-3: control); Hierarchical clustering clearly separated SLE (blue bar) and normal (red bar) samples. Expression values are represented in shades of red and green, indicating expression above and below the median expression value across all samples, respectively.

Hierarchical clustering showed that there were significant differences in the plasma levels of lncRNAs and mRNAs between the 24 new-onset SLE patients and the 12 HCs (Figure 1). According to the criteria of a fold change ≥2 and a *p*-value ≤0.05, 1315 differentially expressed lncRNAs (743 upregulated and 572 downregulated) and 1363 differentially expressed mRNAs (745 upregulated and 618 downregulated) were identified. 

### 3.3. Selection of Candidate lncRNAs and Individual qRT-PCR Confirmation

Candidate lncRNAs identified from the microarray were validated by qRT-PCR according to the following criteria: (1) when compared with HCs, both SLE patients with and without LN had aberrant expression of the candidate lncRNAs with a fold change ≥2 and a *p*-value ≤0.01; (2) the lncRNAs were acquired from the GENCODE, RefSeq, and UCSC Known Gene databases; (3) average raw signal intensity >20; and (4) increased/decreased expression of lncRNAs with a fold change ≥10 in both SLE patients with and without LN. 

Ten lncRNAs met the criteria: ENST00000450640 (lnc0640), ENST00000583643 (lnc3643), ENST00000584688 (lnc4688), ENST00000425150 (lnc5150), ENST00000506655 (lnc6655), NR_027074 (lnc7074), NST00000507514 (lnc7514), ENST00000458228 (lnc8228), ENST00000519603 (lnc9603), and uc001agf.1 (lncagf.1) (Table S2, Supplementary Materials). These 10 lncRNAs were verified in individual samples in the screening phase by qRT-PCR. Of the 10 lncRNAs examined, lnc8228 was not detected in the plasma of the 24 SLE patients and the 12 HCs, whereas the expression of lnc4688 and lnc9603 did not differ significantly between the two groups. The remaining seven lncRNAs showed aberrant expression in the plasma of patients with SLE. Compared with HCs, the expression levels of lnc0640, lnc3643, lnc5150, lnc6655, lnc7514, and lncagf.1 were increased, whereas lnc7074 expression was decreased in all SLE patients (Table 3). 

### 3.4. Independent Validation of Differentially Expressed Plasma lncRNAs

To verify the expression of these seven lncRNAs and to evaluate their potential value as biomarkers for SLE and LN, we performed a qRT-PCR assay in an independent validation phase including a training set and a testing set. A set of plasma lncRNAs (GAS5, linc0597, and lnc-DC) with significantly different expression between SLE patients and HCs was also included, because our previous study found that they may serve as novel non-invasive biomarkers for SLE and LN [26]. 

**Figure 2:**
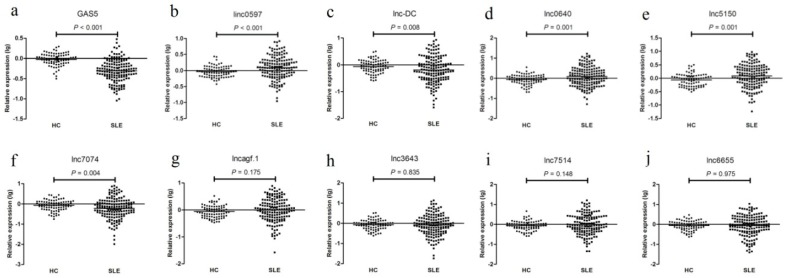
The expression levels of (**a**) GAS5, (**b**) linc0597, (**c**) lnc-DC, (**d**) lnc0640, (**e**) lnc5150, (**f**) lnc7074, (**g**) lncagf.1, (**h**) ln3643, (**i**) lnc7514, and (**j**) lnc6655 between systemic lupus erythematosus (SLE) patients and healthy controls in the training set.

As shown in Figure 2, in the training set, the expression levels of GAS5, linc0597, lnc-DC, lnc0640, lnc5150, lnc7074, and lncagf.1 were consistent with the results from the preliminary validation phase or as described previously [26]. linc0597, lnc0640, and lnc5150 were also increased in all SLE patients compared with those in the HCs, whereas the levels of GAS5, lnc-DC, and lnc7074 were decreased. However, unlike the preliminary validation phase, the levels of lnc3643, lnc6655, lnc7514, and lncagf.1 were not significantly different between SLE patients and HCs.

When the 160 SLE patients were divided into 63 with and 97 without LN, the levels of lnc-DC were significantly higher in LN compared with SLE without nephritis (*p* = 0.005), but no significant difference in levels of GAS5 (*p* = 0.239) and linc0597 (*p* = 0.252) was found between LN and SLE without nephritis, which is in agreement with what we observed previously [26]. The levels of lnc-DC, lnc0640, lnc3643, lnc6655, lnc7074, and lnc7514 were higher in SLE patients with LN compared with those without LN; however, no significant difference in the levels of GAS5, linc0597, lnc5150, and lncagf.1 was found between the two groups (Figure S1, Supplementary Materials). Therefore, lncagf.1 was excluded from the testing set.

**Figure 3:**
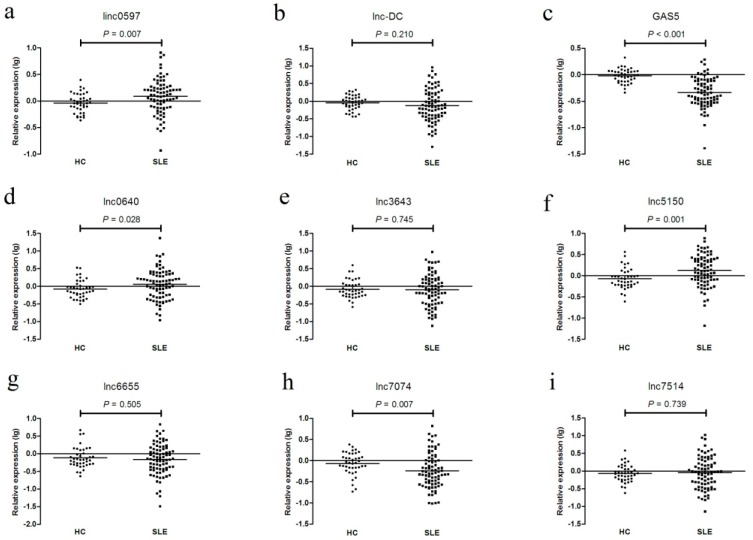
The expression levels of (**a**) linc0597, (**b**) lnc-DC, (**c**) GAS5, (**d**) lnc0640, (**e**) lnc3643, (**f**) lnc5150, (**g**) lnc6655, (**h**) lnc7074, and (**i**) lnc7514 between systemic lupus erythematosus (SLE) patients and healthy controls in the testing set.

In the testing set, as shown in Figure 3 and Figure S2 (Supplementary Materials), the expression levels of GAS5, linc0597, lnc0640, lnc5150, lnc7074, lnc3643, lnc6655, and lnc7514 were consistent with the results from the training set; however, the levels of lnc-DC did not differ between SLE patients and healthy controls, but they did differ between SLE no LN patients and LN patients. 

In the combined set, as shown in Figures S3 and S4 (Supplementary Materials), the expression levels of GAS5, linc0597, lnc-DC, lnc0640, lnc7074, lnc3643, lnc6655, and lnc7514 were consistent with the results from the training set in all comparisons; however, the levels of lnc5150 were significantly higher in SLE patients with LN compared with those patients without LN. To further investigate the stability of lncRNA expression in plasma of SLE patients, we analyzed the potential influence of disease activity and treatment on their expression level, but found no significant difference (Tables S3–S7, Supplementary Materials).

### 3.5. Identification of lncRNAs in Plasma as Potential Biomarkers for SLE

**Figure 4:**
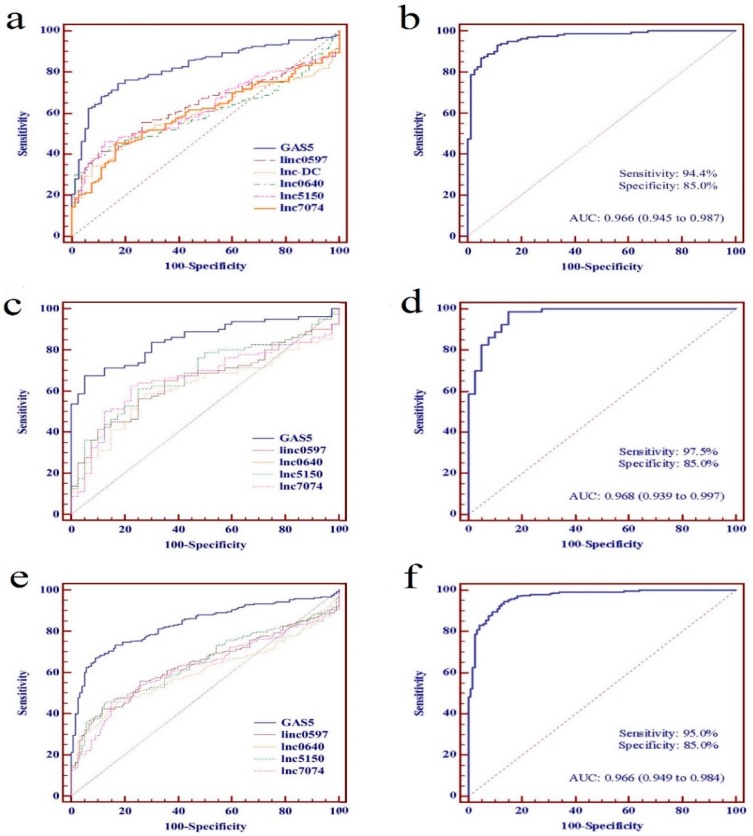
Performance of individual differentially expressed long non-coding RNAs (lncRNAs) and five-lncRNA panel as biomarkers for systemic lupus erythematosus (SLE). (**a**) The receiver operating characteristic (ROC) curves of individual differentially expressed lncRNAs in training set; (**b**) The ROC curve of five-lncRNA panel in training set; (**c**) The ROC curves of individual differentially expressed lncRNAs in testing set; (**d**) The ROC curve of five-lncRNA panel in testing set; (**e**) The ROC curves of individual differentially expressed lncRNAs in combined set; (**f**) The ROC curve of five-lncRNA panel in combined set.

To evaluate further the potential value as SLE biomarkers of the identified lncRNAs individually or as a panel, ROC curve analyses were performed on data from the training set, the testing set, and the combined set, respectively. From the data of the training set, the AUC values of linc0597, GAS5, lnc-DC, lnc0640, lnc5150, and lnc7074 were 0.634 (95% CI: 0.566–0.703), 0.824 (95% CI: 0.771–0.877), 0.597 (95% CI: 0.527–0.667), 0.598 (95% CI: 0.529–0.668), 0.625 (95% CI: 0.556–0.695), and 0.602 (95% CI: 0.531–0.672), respectively (Figure 4a). 

On the basis of the data from the training set, a discrimination model of an lncRNA panel was constructed to predict the probability of a diagnosis with SLE by stepwise logistic regression, and validated in the testing set. The discrimination model of the lncRNA panel was constructed as follows: logit (P = SLE) = −2.594 + 7.503 × linc0597 − 14.253 × GAS5 + 2.853 × lnc5150 − 7.012 × lnc7074 + 6.233 × lnc0640. A patient was classified as “SLE” if logit (P) > 0 according to the logistic regression model, and as “HC” if logit (P) < 0. The AUC value of the panel of five lncRNAs was 0.966 (95% CI: 0.945–0.987) (Figure 4b). From the data of the testing set, the AUC values of these five lncRNAs (linc0597, GAS5, lnc0640, lnc5150, and lnc7074) were 0.649 (95% CI: 0.551-0.746), 0.851 (95% CI: 0.784-0.917), 0.615 (95% CI: 0.514-0.715), 0.683 (95% CI: 0.587-0.779), and 0.662 (95% CI: 0.563-0.760), respectively (Figure 4c). The AUC of the panel of five lncRNAs was 0.968 (95% CI: 0.939-0.997) (Figure 4d). 

We further evaluated the identified lncRNAs as biomarkers for SLE individually or as a panel in a combined set including 240 patients with SLE and 120 HCs. The AUC values of linc0597, GAS5, lnc0640, lnc5150, and lnc7074 were 0.640 (95% CI: 0.584-0.696), 0.833 (95% CI: 0.791-0.874), 0.604 (95% CI: 0.547-0.661), 0.645 (95% CI: 0.589-0.701), and 0.622 (95% CI: 0.565-0.679), respectively (Figure 4e), and the AUC value was 0.966 (95% CI: 0.949-0.984) when the panel of five lncRNAs was evaluated, which was also significantly higher than the AUC value of GAS5 (Figure 4f). 

Finally, the 240 SLE patients were divided into 95 with and 145 without LN. On the basis of the data from the independent validation phase, a discrimination model of the lncRNA panel was also constructed to predict the probability of an LN diagnosis. Stepwise logistic regression and ROC curve analysis, respectively, were used to explore the potential value of the differentially expressed lncRNAs as novel biomarkers to distinguish SLE with and without LN both individually and as a panel. The AUC values are shown in Table 4. The stepwise logistic regression model showed that lnc-DC, lnc5150, and lnc7514 could be used as a panel of biomarkers to distinguish SLE with LN from SLE without LN; no other panels with statistical significance were observed. The discrimination model of the panel of three lncRNAs was constructed to predict the probability of diagnosis with LN as follows: logit (P = LN) = 0.013 + 1.706 × lnc-DC − 2.053 × lnc5150 + 1.426 × lnc7514, which generated an AUC of 0.725 (95% CI: 0.659-0.791) (Table 4); however, it was not significantly different compared with the AUC value of lnc-DC or lnc7514.

### 3.6. Plasma Levels of lncRNAs in Patients with SLE, RA, and pSS

**Figure 5:**
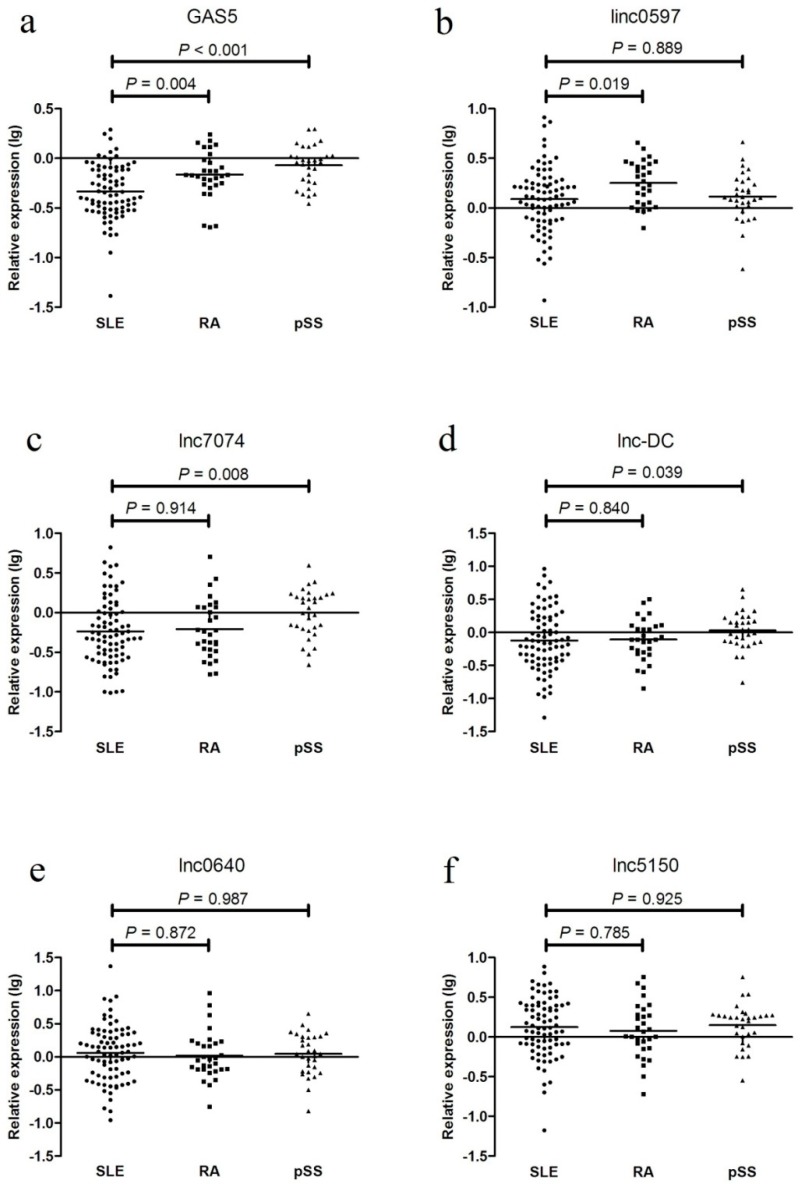
Validation of differentially expressed long non-coding RNAs (lncRNAs) in systemic lupus erythematosus (SLE) and disease controls (rheumatoid arthritis (RA) patients and primary Sjögren’s syndrome (pSS) patients). (**a**) GAS5; (**b**) linc0597; (**c**) lnc7074; (**d**) lnc-DC; (**e**) lnc0640; (**f**) lnc5150.

In the external validation phase, the levels of these five differentially expressed lncRNAs were examined in 30 patients with RA and 31 patients with pSS. GAS5 expression was decreased in the SLE patients from the testing set compared with that in the RA and pSS patients; linc0597 levels were lower in the SLE patients from the testing set compared with those in the RA patients; lnc7074 levels were lower in the SLE patients from the testing set compared with those in the pSS patients; and lnc-DC levels were lower in the SLE patients compared with those in the pSS patients. No significant difference was found between the SLE and RA patients. Compared with the SLE patients, no significant differences in the levels of lnc0640 and lnc5150 were found in the RA and pSS patients (Figure 5). 

**Figure 6:**
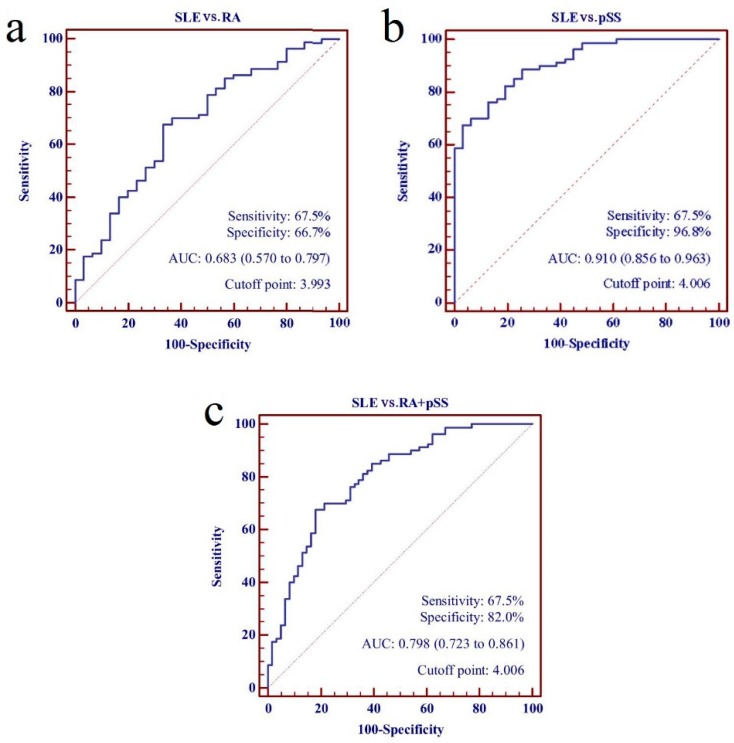
Performance of five- long non-coding RNAs (lncRNAs) panel as biomarkers for discriminating systemic lupus erythematosus (SLE) from rheumatoid arthritis (RA) patients and primary Sjögren’s syndrome (pSS) patients. (**a**) SLE vs. RA; (**b**) SLE vs. pSS; (**c**) SLE vs. RA + pSS.

Subsequently, the panel of five lncRNAs from the training set was assessed further in the SLE patients from the testing set and all disease controls, which generated an AUC value for the risk score of 0.798 (95% CI: 0.723–0.861). The risk score also significantly discriminated the patients with SLE in the testing set from the RA and the pSS patients, with AUC values of 0.683 (95% CI: 0.570–0.797) and 0.910 (95% CI: 0.856–0.963), respectively (Figure 6). 

### 3.7. KEGG Pathway Analysis

KEGG pathway analysis identified 11 pathways that were associated with the upregulated mRNAs and 4 pathways related to the downregulated mRNAs. The “MAPK signaling pathway—*Homo sapiens* (human)”, “PPAR signaling pathway—*Homo sapiens* (human)”, and “glycosphingolipid biosynthesis—lacto and neolacto series—*Homo sapiens* (human)” were the top three pathways for the upregulated protein-coding genes, whereas the most enriched network was “axon guidance—*Homo sapiens* (human)” for the downregulated protein-coding genes (Figure S5, Supplementary Materials). 

### 3.8. Construction of a Co-Expression Network 

Subsequently, in combination with the validation results by qRT-PCR and the data from the LncRNA Microarray, we established lncRNA–mRNA co-expression network analysis to explore the biological functions of the differentially expressed lncRNAs in SLE. The lncRNA–mRNA co-expression network analysis revealed that GAS5, lnc0640, lnc5150, lnc7074, lnc3643, lnc6655, and lnc7514 were correlated highly with 174, 18, 48, 30, 36, 28, and 20 mRNAs, respectively. The network also indicated that the target genes of GAS5, lnc0640, and lnc5150 were dual specificity phosphatase 4 (DUSP4), arrestin beta 2 (ARRB2), and ribosomal protein S6 kinase A5 (RPS6KA5), respectively, indicating that the contribution of GAS5, lnc0640, and lnc5150 to the pathogenesis of SLE may be through the MAPK signaling pathway (Figures S6 and S7, Supplementary Materials). 

### 3.9. Construction of a ceRNA Network 

According to ceRNA hypothesis, lncRNAs may act as a “sponge” of miRNA competing with the miRNA response elements (MREs), thereby regulating miRNA-mediated biological processes [34,35]. To explore the underlying mechanism of the differentially expressed lncRNAs validated by qRT-PCR in the pathogenesis of SLE, we used ceRNA analysis in SLE to construct the regulatory network of mRNA–miRNA–lncRNA. The ceRNA analysis pointed out that GAS5, lnc0640, lnc3643, lnc6655, and lnc7074 could act as ceRNAs to regulate the expression of the predicted target genes by competing for common miRNA-binding sites with mRNAs (Figure S8).

## 4. Discussion

In the present study, we investigated the expression profile of plasma lncRNAs in SLE patients, and found that five lncRNAs (linc0597, GAS5, lnc0640, lnc5150, and lnc7074) were aberrantly expressed as compared with HCs. Then, we evaluated the value of these five lncRNAs as potential biomarkers for SLE. Finally, we explored the potential targets and mechanism of aberrantly expressed lncRNAs using bioinformatics.

During the preliminary screening stage of the present study, 10 differentially expressed candidate lncRNAs were selected for preliminary validation and independent validation. Compared with HCs, the plasma levels of lnc0640 and lnc5150 were significantly elevated, whereas lnc7074 expression was significantly decreased. In accordance with our previous studies, linc0597 and GAS5 were differentially expressed in both the training and testing sets; however, there was no significant change in lnc-DC expression in the testing set, which may be due to the small sample size.

On the basis of the results of independent validation with a large sample size, we investigated the plasma levels of lncRNAs in SLE patients with and without LN in both training and testing sets. The expression levels of lnc-DC, lnc0640, lnc3643, lnc5150, lnc6655, lnc7074, and lnc7514 were significantly higher in the patients with LN compared with those without LN, suggesting that the plasma levels of these seven lncRNAs may reflect kidney pathology in SLE.

Our recent study suggested that plasma levels of linc0597 and GAS5 may serve as potential biomarkers for SLE; lnc-DC could serve as a biomarker for distinguishing SLE with LN from SLE without LN [26]. Available evidence has indicated that circulating lncRNAs are stable in the plasma, because they are resistant to digestion from endogenous RNases [31,36,37,38]. In addition, a large number of studies have demonstrated that lncRNAs may serve as biomarkers for the diagnosis of cancer, cardiovascular diseases, among others. [22,23,24,25]. Therefore, we evaluated the value of these differentially expressed lncRNAs as biomarkers for SLE. Our results showed that GAS5, lnc7074, linc0597, lnc0640, and lnc5150 could be potential biomarkers for SLE. According to the expression of lncRNAs in RA and pSS patients, GAS5, lnc7074, and linc0597 demonstrated better specificity than the other two lncRNAs. The combination of these five lncRNAs in a logistic regression model demonstrated a higher AUC value than when they were used individually. Additionally, the combination of these five lncRNAs could distinguish SLE from RA and pSS. We investigated the value of lnc-DC, lnc0640, lnc3643, lnc5150, lnc6655, lnc7074, and lnc7514 as biomarkers for LN individually and as a panel, and found that the panel of lnc-DC, lnc5150, and lnc7514 did not significantly increase the AUC value compared with when they were used individually.

Currently, the function of most lncRNAs and their mechanism in disease pathogenesis are not clear. Therefore, we explored the potential targets and mechanism of the differentially expressed lncRNAs by bioinformatics. KEGG analysis demonstrated the association of the MAPK signaling pathway with SLE. It has been confirmed that the MAPK signaling pathway can regulate the immune response of T cells and B cells as well as the production of multiple SLE-related inflammatory factors, such as TNF-α, IL-1/6, and IFN [39,40]. Molad et al. reported that the activity of two members of the MAPK family, namely, ERK and JNK, are associated with SLE disease activity [40]. lncRNA–mRNA co-expression network analysis showed that GAS5, lnc0640, and lnc5150 may participate in the development of SLE through the MAPK signaling pathway; DUSP4 (also called MKP2) may be a positively regulated target gene of GAS5, ARRB2 may be a positively regulated target gene of lnc0640, and RPS6KA5 (also called MSK1) may be a positively regulated target gene of lnc5150. Furthermore, we predicted the target genes of the lncRNAs and their regulatory mechanism, and the results showed that GAS5, lnc0640, lnc3643, lnc6655, and lnc7074 could act as ceRNAs to regulate the expression of the predicted target genes by acting as “sponges” for the target miRNAs. Increasing evidence has shown that lncRNAs can function as ceRNAs in distinct physiological and pathophysiological states. Many validated ceRNA pairs participate in the initiation and progression of cancers, and systemic ceRNA network analyses revealing the potential of ceRNAs in diagnosis, therapy, and prognosis of cancers have also been performed [41]. Currently, the exact role of the ceRNA network in SLE remains undefined, and further studies are awaited to experimentally validate the true ceRNAs for certain non-coding transcripts and provide functional information for these non-coding RNAs. Dissecting the ceRNA network in SLE is likely to enhance our understanding of the roles of transcriptome, particularly non-coding transcripts, and enrich our knowledge of the pathogenesis, diagnosis, and therapy of this disease.

Nevertheless, several limitations should be noted in the present study. First, all the study subjects were recruited from two tertiary hospitals, and the sample size was somewhat small, which may restrict the generalizability of our results. Second, it would be beneficial to know the prediction accuracy that would be obtained when comparing the model with clinical data and with clinical data plus a biomarker panel. However, since most clinical parameters (rheumatoid factor, anti-CCP antibodies, anti-Ro/La, anti-dsDNA, etc.) were not available for healthy controls, we could thus not compare the model with clinical data and with clinical data plus a biomarker panel. Therefore, further studies with a large sample size in different populations are needed to confirm our findings.

## 5. Conclusions

Taken together, the plasma levels of the panel of five lncRNAs (GAS5, lnc7074, linc0597, lnc0640, and lnc5150) may serve as biomarkers for SLE. GAS5, lnc0640, and lnc5150 may participate in the development of SLE through the MAPK signaling pathway. In addition, GAS5, lnc0640, lnc3643, lnc6655, and lnc7074 could act as ceRNAs to regulate the expression of the predicted target genes by acting as “sponges” for the target miRNAs. Future studies are needed to further unveil the exact role of these lncRNAs and their ceRNA network in SLE.

## Figures and Tables

**Table 1:** Basic features of systemic lupus erythematosus (SLE) patients and healthy controls in large-scale independent validation phase ^a^.

Characteristics	Training Set	Testing Set
**Control subjects**	**80**	**40**
Age (year)	37 ± 14	34 ± 12
Sex (female/male)	74/6	37/3
**Patients with SLE**	**160**	**80**
Age (year)	38 ± 14	38 ± 12
Sex (female/male)	148/12	74/6
Disease duration (year)	4 (1, 9)	3 (0, 9)
SLEDAI-2K	12 (8, 19)	13 ± 8
Disease manifestations		
Renal disease (%)	63 (39)	32 (40)
Vasculitis (%)	6 (4)	4 (5)
Arthritis (%)	64 (40)	26 (33)
Myositis (%)	12 (8)	4 (5)
Rash (%)	77 (48)	35 (44)
Alopecia (%)	67 (42)	26 (33)
Oral ulcer (%)	30 (19)	11 (14)
Pleuritis (%)	18 (11)	8 (10)
Leukopenia (%)	25 (16)	21 (26) ^*^
Thrombocytopenia (%)	43 (27)	19 (24)
Fever (%)	45 (28)	20 (25)
Nervous system disorder (%)	41 (26)	18 (23)
Low complement (%)	108 (68)	52 (65)
Autoantibodies		
Anti-dsDNA (%)	71 (44)	27 (34)
Anti-Sm (%)	53 (34)	26 (33)
Anti-SSA (%)	112 (71)	55 (71)
Anti-SSB (%)	26 (17)	14 (18)
Anti-RNP (%)	64 (41)	33 (42)
Anti-Ribosomal P (%)	48 (31)	24 (31)
Medical therapy		
Prednisone dose ≥15 mg/day (%)	82 (51)	42 (52)
Prednisone dose <15 mg/day (%)	78 (49)	38 (48)
Antimalarials (%)	127 (79)	57 (71)
Azathioprine, MTX, or CTX (%)	31 (19)	11 (14)

^a^ Normally distributed data were expressed as means ± standard deviation (SD), variables with a skewed distribution were presented as median (interquartile range). Categorical variable values were described as number (%). SLEDAI-2K: Systemic Lupus Erythematosus Disease Activity Index 2000; dsDNA: double-stranded DNA; Sm: Smith; SSA: Sjögren’s syndrome-related antigen A; SSB: Sjögren’s syndrome-related antigen B; RNP: Ribonucleoprotein; MTX: methotrexate; CTX: cyclophosphamide; ^*^ *p* ≤ 0.05.

**Table 2:** Basic features of disease controls (rheumatoid arthritis (RA) patients and primary Sjögren’s syndrome (pSS) patients) ^a^.

Characteristics	RA	pSS
Age (year)	53.47 ± 1.63	47.39 ± 9.96
Sex (female/male)	20/10	31/0
Disease duration (year)	7 (3, 19)	5.74 ± 4.40
Medical therapy		
Prednisone (%)	20 (67)	27 (87)
Antimalarials (%)	9 (30)	24 (77)
TGP (%)	12 (40)	22 (71)
Leflunomide, MTX, or NSAID (%)	28 (93)	8 (26)

^a^ Normally distributed data were expressed as means ± standard deviation (SD), variables with a skewed distribution were presented as median (interquartile range). Categorical variable values were described as number (%). TGP: total glucosides of paeony; MTX: methotrexate; NSAID: nonsteroidal anti-inflammatory drug.

**Table 3:** The expressions of 10 candidate long non-coding RNAs (lncRNAs) in the preliminary validation phase.

lncRNAs	Healthy Controls (*n* = 12)	SLE Patients (*n* = 24)	*Z*	*P*
lnc0640	1.00 (0.68, 1.27)	4.52 (3.46, 5.29)	−4.832	<0.001
lnc3643	0.97 (0.79, 1.23)	2.69 (1.95, 5.53)	−4.799	<0.001
lnc5150	0.74 (0.59, 1.64)	3.01 (1.53, 3.94)	−3.893	<0.001
lnc6655	1.05 (0.74, 1.27)	8.35 (5.67, 10.2)	−4.832	<0.001
lnc7074	1.00 (0.69, 1.32)	0.24 (0.16, 0.29)	−4.832	<0.001
lnc7514	0.71 (0.56, 0.89)	1.69 (1.46, 2.12)	−3.356	<0.001
lncagf.1	0.74 (0.44, 1.68)	2.01 (1.21, 2.95)	−3.020	0.002
lnc4688	1.05 (0.63, 1.16)	0.93 (0.62, 1.27)	−0.201	0.856
lnc9603	0.92 (0.68, 1.34)	0.90 (0.62, 1.06)	−0.470	0.655

**Table 4:** Performance of individual differentially expressed long non-coding RNAs (lncRNAs) and three-lncRNA panel as biomarkers for lupus nephritis (LN).

lncRNA	AUC	Sensitivity	Specificity	Cutoff Point
lnc-DC	0.683 (0.612–0.753)	0.526	0.807	1.070
lnc0640	0.644 (0.571–0.717)	0.421	0.821	2.250
lnc3643	0.671 (0.600–0.742)	0.526	0.759	1.172
lnc5150	0.601 (0.527–0.675)	0.421	0.772	2.193
lnc6655	0.631 (0.559–0.703)	0.758	0.448	0.607
lnc7074	0.665 (0.594–0.735)	0.632	0.669	0.673
lnc7514	0.684 (0.614–0.755)	0.674	0.662	0.990
three-lncRNA panel	0.725 (0.659–0.791)	0.642	0.766	0.000

AUC: area under curve.

## Supplementary Materials

The following are available online at https://www.mdpi.com/2218-273X/9/6/206/s1. **Figure S1:** The expression levels of GAS5, linc0597, lnc-DC, lnc0640, lnc5150, lnc7074, lncagf.1, ln3643, lnc7514, and lnc6655 between SLE no LN patients and LN patients in the training set; **Figure S2:** The expression levels of linc0597, lnc-DC, GAS5, lnc0640, lnc3643, lnc5150, lnc6655, lnc7074, and lnc7514 between SLE no LN patients and LN patients in the testing set; **Figure S3:** The expression levels of linc0597, lnc-DC, GAS5, lnc0640, lnc3643, lnc5150, lnc6655, lnc7074, and lnc7514 between SLE patients and healthy controls in the combined set; **Figure S4:** The expression levels of linc0597, lnc-DC, GAS5, lnc0640, lnc3643, lnc5150, lnc6655, lnc7074, and lnc7514 between SLE no LN patients and LN patients in the combined set; **Figure S5:** Differentially expressed mRNAs with significant enrichment score of pathway: (**a**) upregulated mRNAs; (**b**) downregulated mRNAs; **Figure S6:** Co-expression network of associated mRNAs with GAS5 (ENST00000449289); **Figure S7:** Co-expression network of associated mRNAs with individual lncRNAs of lnc0640 (ENST00000450640), lnc3643 (ENST00000583643), lnc5150 (ENST00000425150), lnc6655 (ENST00000506655), lnc7074 (NR_027074), and lnc7514 (ENST00000507514); **Figure S8:** ceRNA networks of GAS5(ENST00000449289), lnc0640 (ENST00000450640), lnc3643 (ENST00000583643), lnc6655 (ENST00000506655), and lnc7074 (NR_027074); **Table S1:** Primer sequences used for qPCR; **Table S2:** Ten differential expression plasma candidate lncRNAs with SLE by microarray; **Table S3:** Associations of the expression of plasma lnc7074 with categorical clinical parameters of SLE patients; **Table S4:** Associations of the expression of plasma linc0597 with categorical clinical parameters of SLE patients; **Table S5:** Associations of the expression of plasma lnc0640 with categorical clinical parameters of SLE patients; **Table S6:** Associations of the expression of plasma lnc05150 with categorical clinical parameters of SLE patients; **Table S7:** Associations of the expression of plasma GAS5 with categorical clinical parameters of SLE patients.
