# Supplementary material for: Differential Plasma Expression Profiles of Long Non-Coding RNAs Reveal Potential Biomarkers for Systemic Lupus Erythematosus
Source: Biomolecules. 2019 May 28;9(6):206. doi: 10.3390/biom9060206 (PMC6627908; doi:10.3390/biom9060206)
Supplement: Supplementary file 1 [file biomolecules-09-00206-s001.pdf]

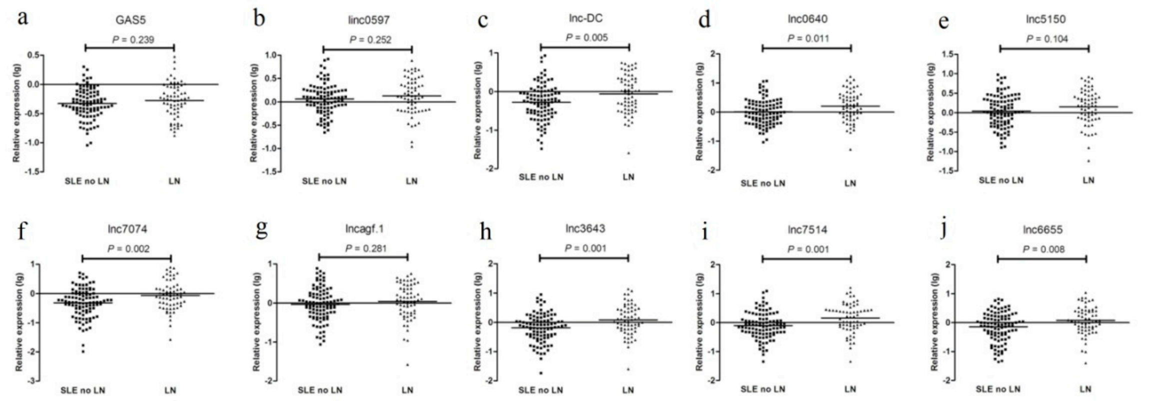

Figure S1 The expression levels of (a) GAS5, (b) linc0597, (c) lnc-DC, (d) lnc0640, (e) lnc5150, (f) lnc7074, (g) lncagf.1, (h) lnc3643, (i) lnc7514 and (j) lnc6655 between systemic lupus erythematosus (SLE) no lupus nephritis (LN) patients and LN patients in the training set.

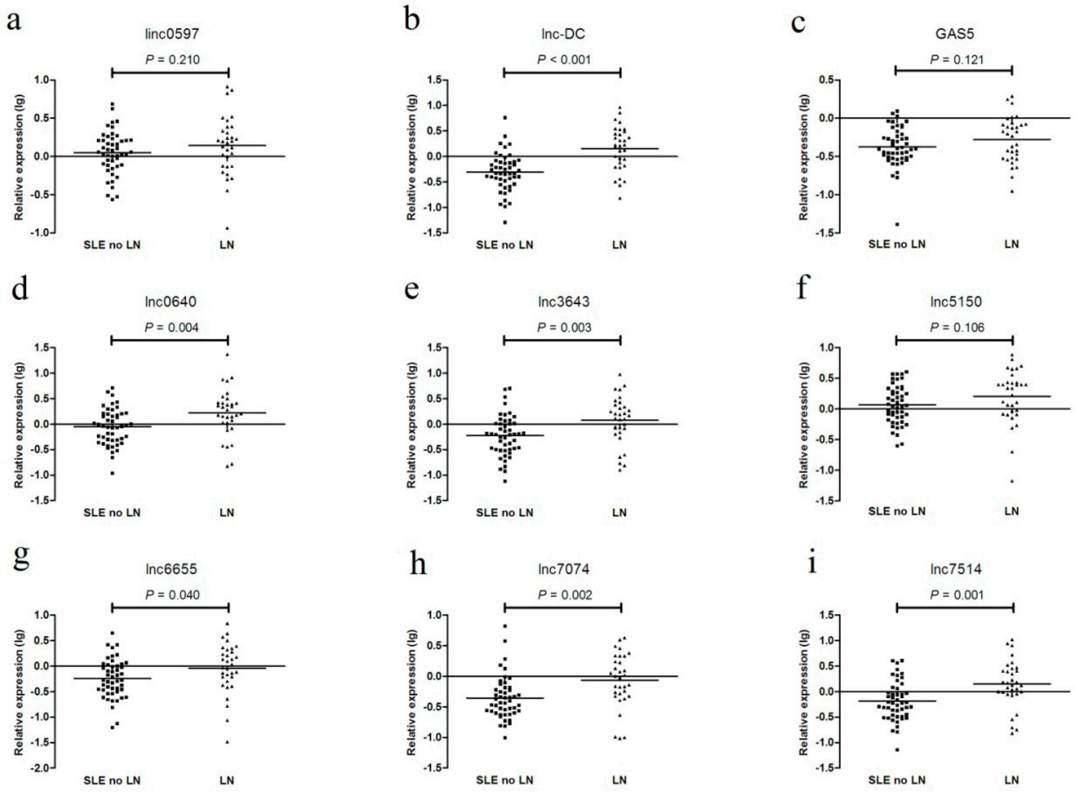

Figure S2 The expression levels of (a) linc0597, (b) lnc-DC, (c) GAS5, (d) lnc0640, (e) lnc3643, (f) lnc5150, (g) lnc6655, (h) lnc7074 and (i) lnc7514 between systemic lupus erythematosus (SLE) no lupus nephritis (LN) patients and LN patients in the testing set.

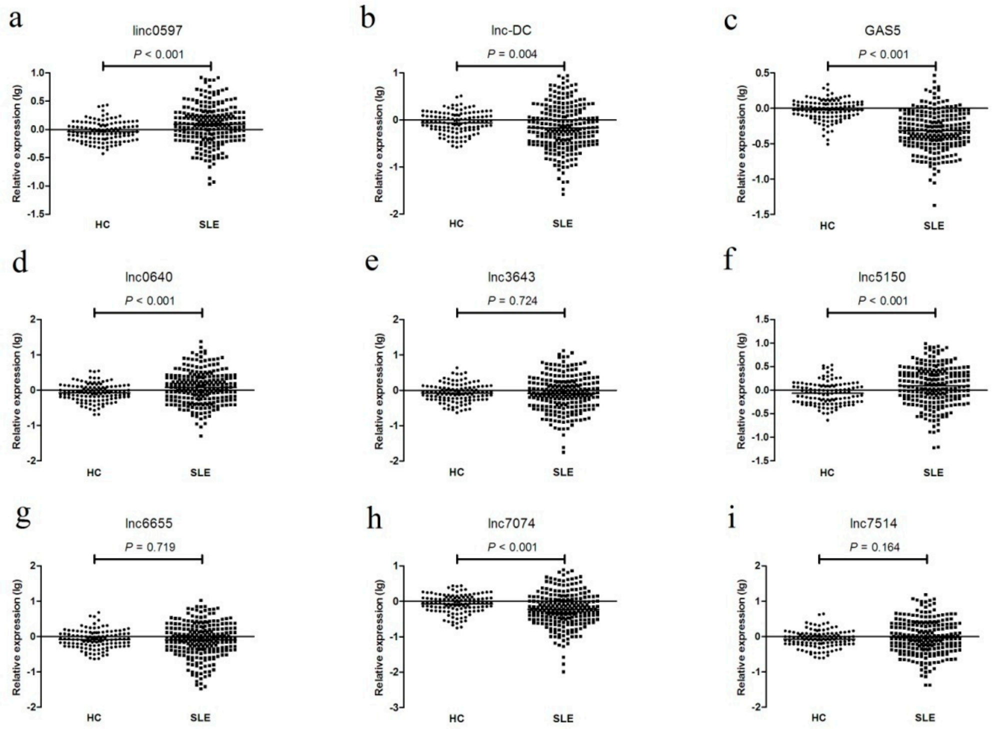

Figure S3 The expression levels of (a) linc0597, (b) lnc-DC, (c) GAS5, (d) lnc0640, (e) lnc3643, (f) lnc5150, (g) lnc6655, (h) lnc7074 and (i) lnc7514 between systemic lupus erythematosus (SLE) patients and health controls in the combined set.

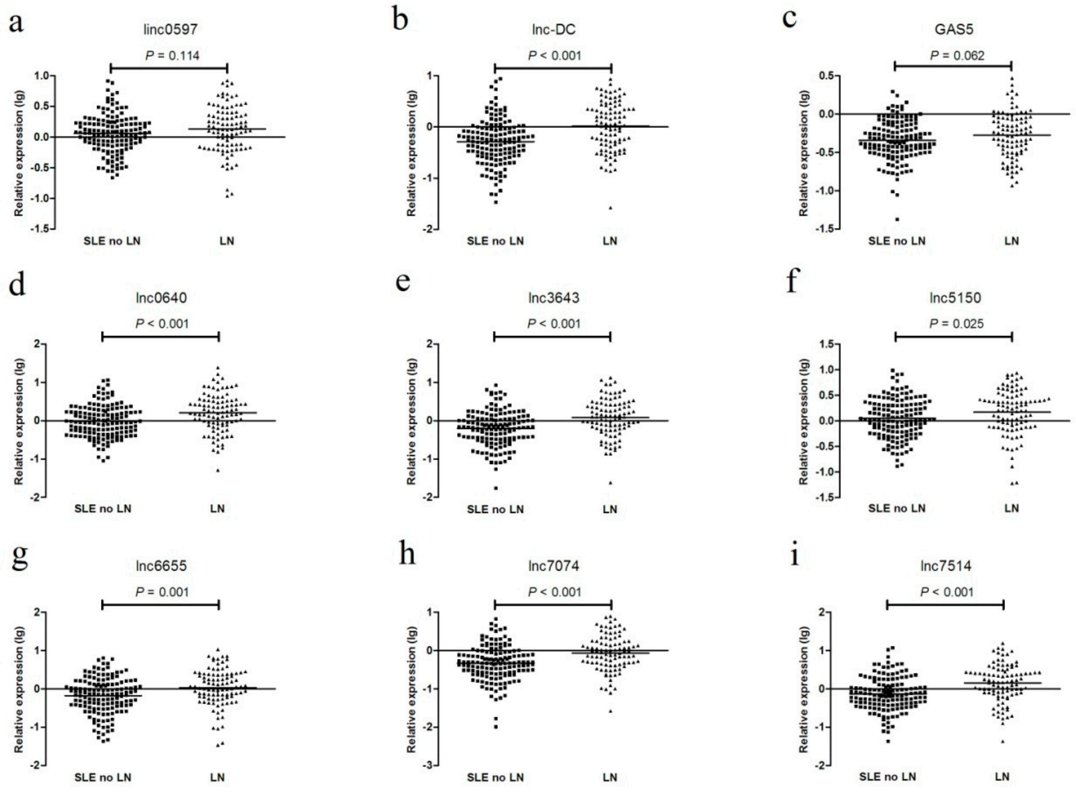

Figure S4 The expression levels of (a) linc0597, (b) lnc-DC, (c) GAS5, (d) lnc0640, (e) lnc3643, (f) lnc5150, (g) lnc6655, (h) lnc7074 and (i) lnc7514 between systemic lupus erythematosus (SLE) no lupus nephritis (LN) patients and LN patients in the combined set.

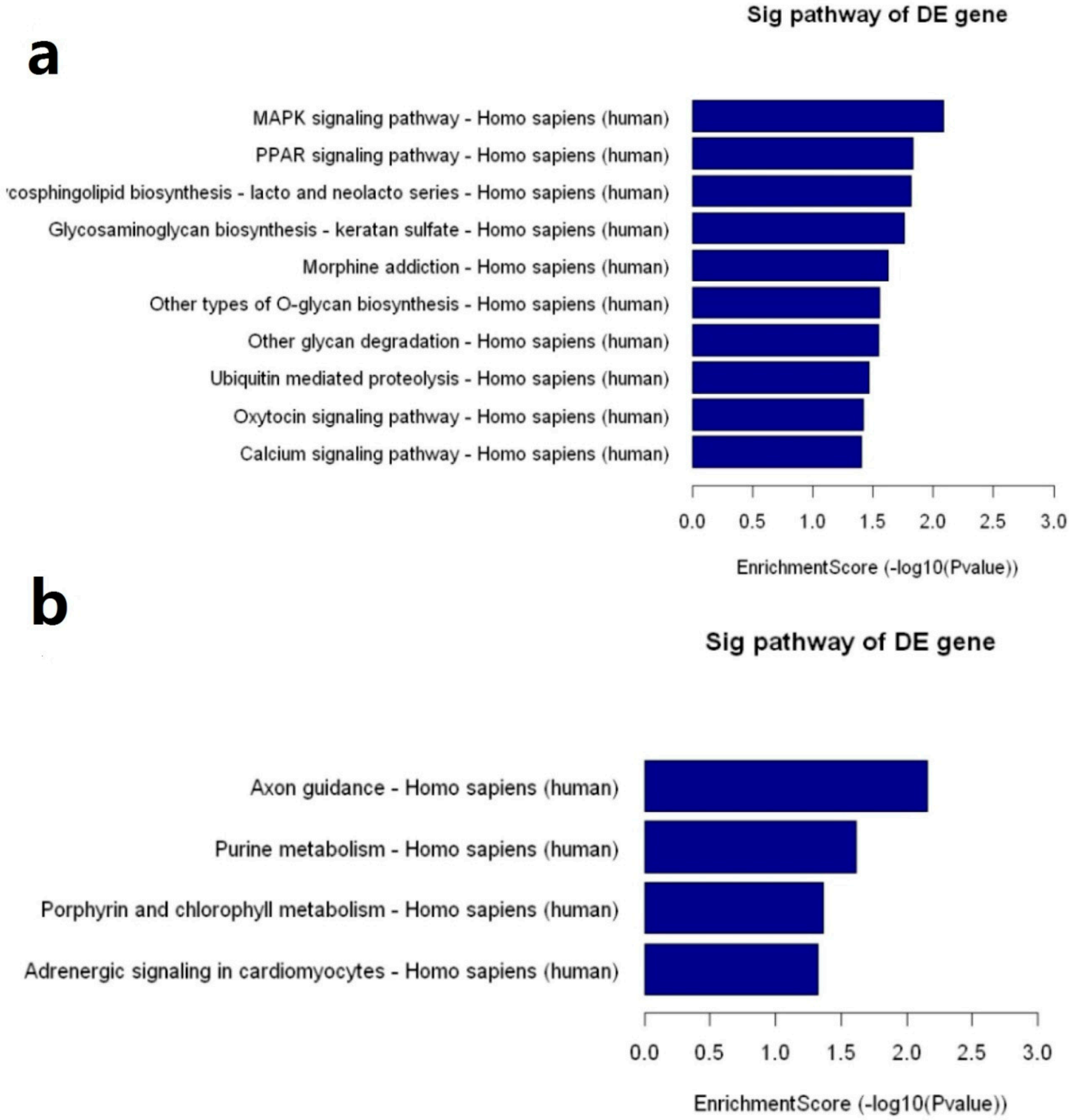

Figure S5 Differentially expressed mRNAs with significant enrichment score of pathway.
(a) up-regulated mRNAs; (b) down-regulated mRNAs

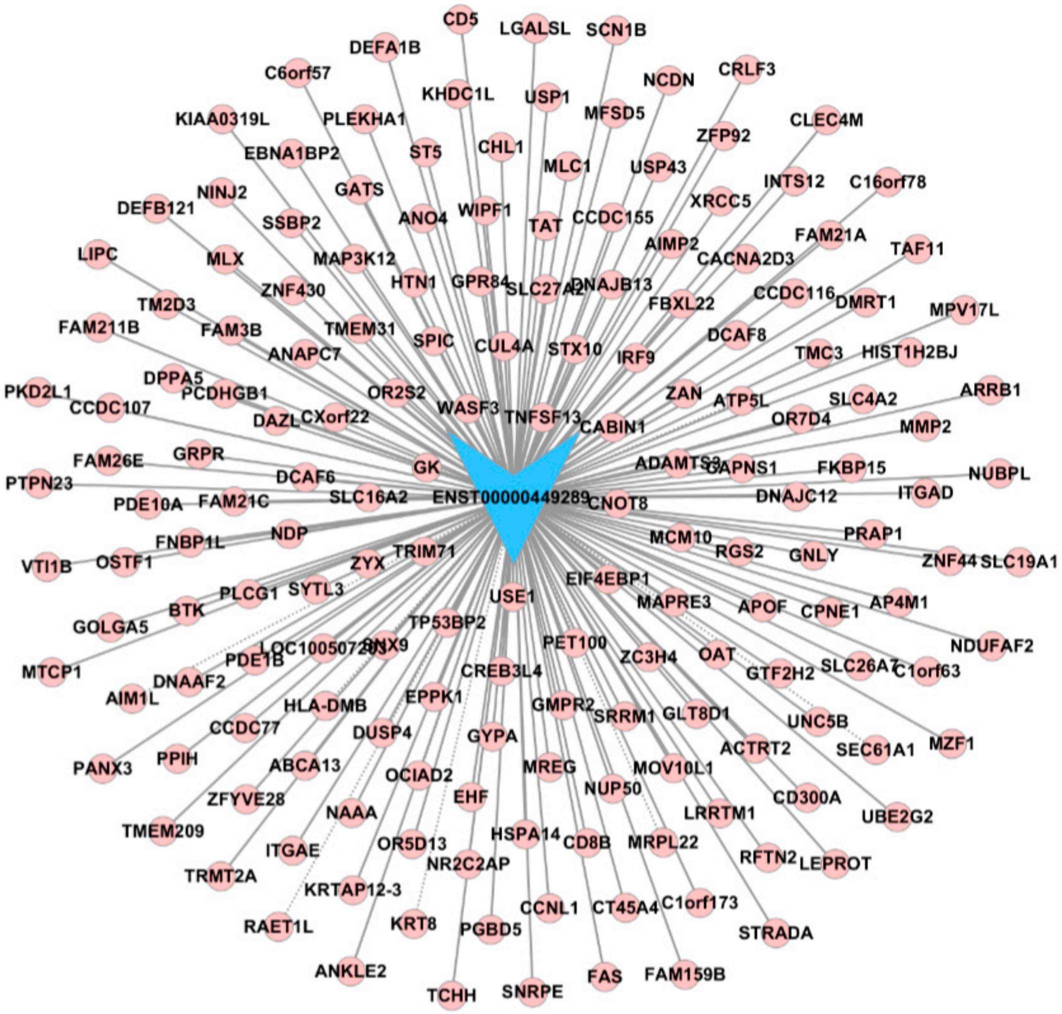

Figure S6 Co-expression network of associated mRNAs with GAS5 (ENST00000449289); Node with round colored in pink are mRNAs; Node with V shape colored in blue are long non-coding RNAs (lncRNAs); Solid lines mean positive correlations while dashed lines mean negative correlations; Node size represents the node degrees

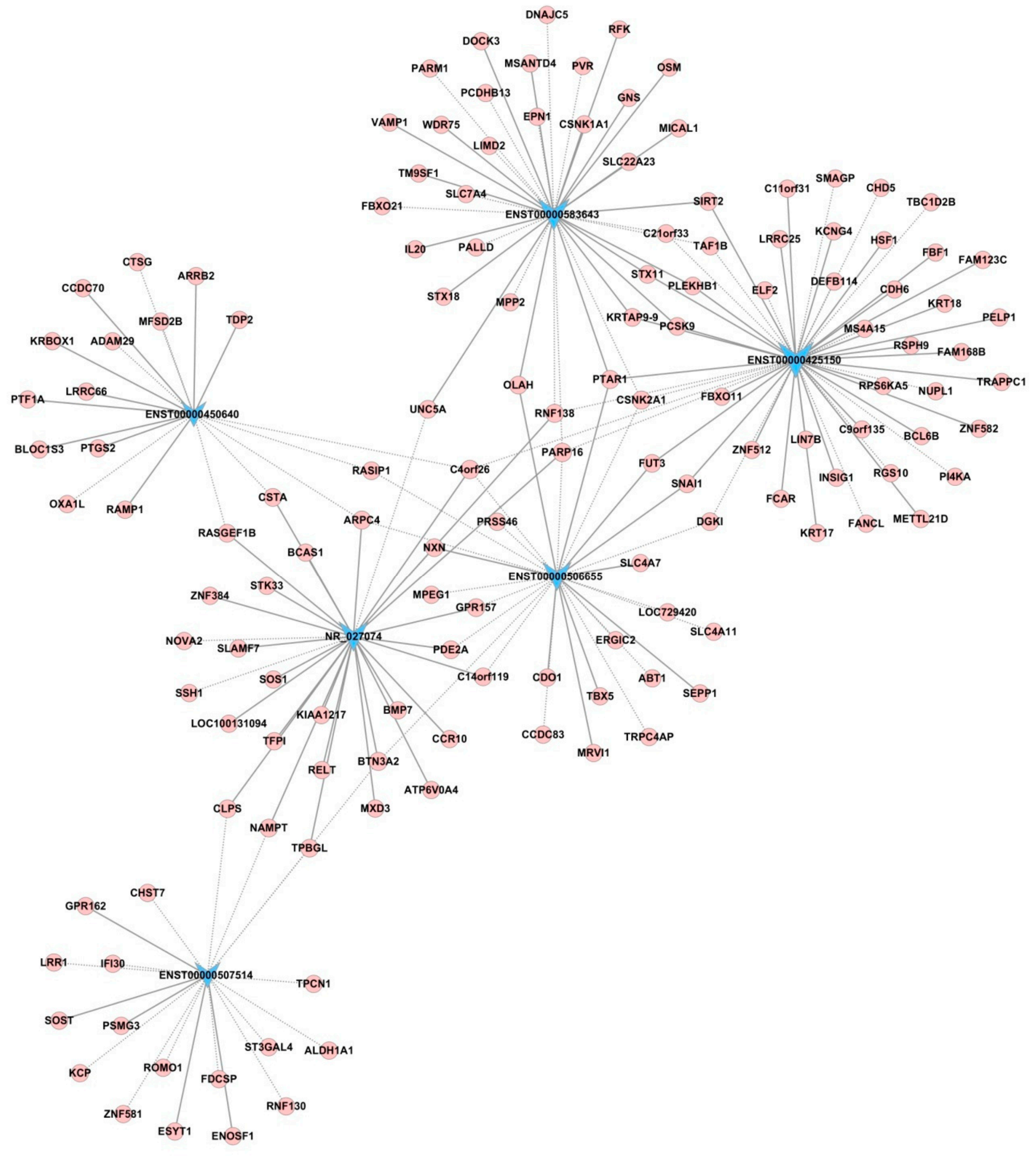

Figure S7 Co-expression network of associated mRNAs with individually lncRNA of lnc0640 (ENST00000450640), lnc3643 (ENST00000583643), lnc5150 (ENST00000425150), lnc6655 (ENST00000506655), lnc7074 (NR_027074) and lnc7514 (ENST00000507514); Node with round colored in pink are mRNAs; Node with V shape colored in blue are long non-coding RNAs (lncRNAs); Solid lines mean positive correlations while dashed lines mean negative correlations; Node size represents the node degrees

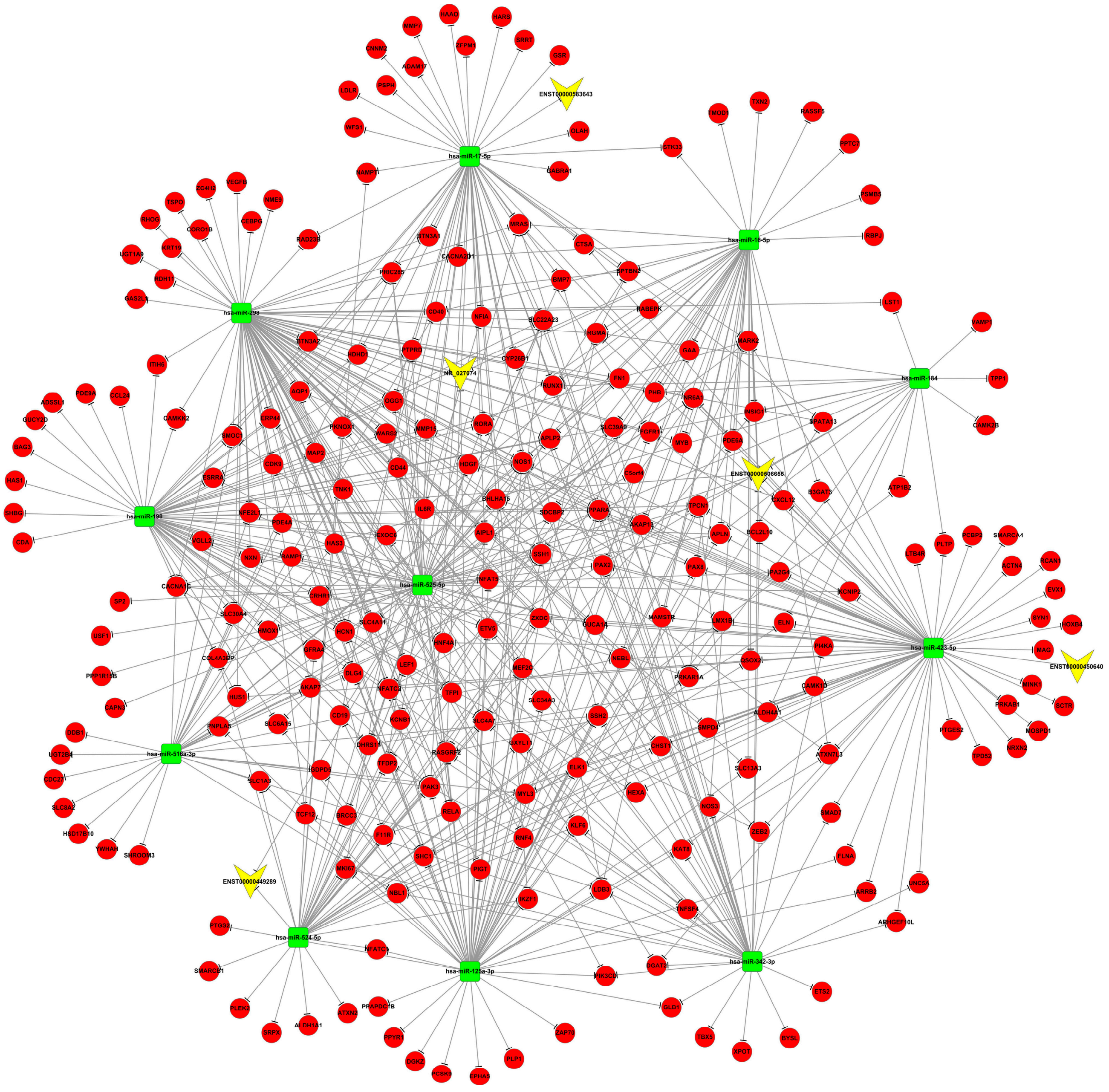

Figure S8 Competing endogenous RNA (ceRNA) networks of GAS5(ENST00000449289), lnc0640 (ENST00000450640), lnc3643 (ENST00000583643), lnc6655 (ENST00000506655) and lnc7074 (NR_027074). Node with round colored in red are mRNAs; Node with V shape colored in yellow are long non-coding RNAs (lncRNAs); Node with square colored in green are microRNAs (miRNAs); The edges represent sequence matching, and miRNAs connects expression correlated lncRNA or mRNA

Table S1 Primers sequences used for qPCR

Gene	Primers [a]	Amplicon (bp)
GAPDH	F:5' GGGAAACTGTGGCGTGAT3'<br>R :5’ GAGTGGGTGTCGCTGTTGA3’	299
linc0597	F:5' TTGGATTCATCCCGTTCACCTCCA 3'<br>R:5' CAGCATGACGATCAAGCGAGATTC 3'	121
lnc-DC	F:5' GATCGTCATCCCTTCCTGG3'<br>R :5’ GAAACAACCCCTCTTCCCTG3’	139
GAS5	F:5' TATGGTGCTGGGTGCGGAT3'<br>R :5’ CCAATGGCTTGAGTTAGGCTT3’	121
lnc0640	F:5' ACAAAGAGGAAGGAACGGCA3'<br>R :5’ AGTGTTGAAGGGTGCAGATGG3’	70
lnc3643	F:5' CTGTTGTCTTGGCCTTGGTG3'<br>R:5' AATGGCAAGCAGGTAGGCTG3'	95
lnc4688	F:5' CCCTCCACCATCACCCAAT3'<br>R:5' GGCAAACCTCTGCTCACCAC3'	146
lnc5150	F:5' ATCTCATTAGTGGACGCCG3'<br>R:5' AGCATCACCGAAAGGAAGC3'	105
lnc6655	F:5' AAACACCCTCGTTCCTCATCC3'<br>R:5' CTTACAGCTCGAATGCCCAG3'	130
lnc7074	F:5' AAGACCCTCCCTTGAACCGT3'<br>R:5' TCAAACCCTGTGGGGCTTTC3'	153
lnc7514	F:5' AAAGCGGCTGTGGCTGAGAA3'<br>R:5' CTGGAGGAAGGTGGTGTGTAG3'	61
lnc8228	F:5' AGTGCCCCCCACAATCGTA3'<br>R:5' GGAATGGCGACCTAAACACC3'	236
lnc9603	F:5' GAGACTGTCAGGAGTTGGTGG3'<br>R:5' ATCGTGGTGAGGCTGTTGC3'	234
lncagf.1	F:5' GTACCTGCCCATACCGATG3'<br>R:5' GGTTCCTTGCGAAGTCTCA3'	129

[a] F: Forward; R: Reverse

Table S2 Ten differential expression plasma candidate long non-coding RNAs (lncRNAs) with systemic lupus erythematosus (SLE) by microarray [a]

Seqname	SLE no LN vs. HC			LN vs. HC			Gene Symbol	Source	Raw signal		
	P-value	FC	Regulation	*P*-value	FC	Regulation			SLE no LN	LN	HC
lnc7074	9.15E-04	105.13	down	1.87E-04	96.18	down	LOC283761	RefSeq	57.13	172.52	6499.61
lnc4688	4.50E-03	46.77	down	1.99E-03	77.43	down	RP11-40A13.1	GENCODE	70.76	143.04	3647.42
lnc8228	1.78E-03	43.91	down	6.24E-04	10.16	down	RP11-429G19.2	GENCODE	23.04	341.31	1257.53
lnc3643	8.16E-03	48.63	up	5.34E-03	24.03	up	RP11-720N19.2	GENCODE	3565.07	6476.19	127.98
lnc0640	4.63E-03	11.03	up	4.31E-04	18.02	up	RP5-1022P6.4	GENCODE	1821.59	14578.98	317.14
lnc7514	1.30E-03	14.37	up	9.81E-03	14.41	up	CTD-2384B11.2	GENCODE	3603.55	7888.23	238.44
lnc6655	4.15E-04	141.71	up	1.39E-03	45.41	up	RP11-143A12.3	GENCODE	3323.25	7411.13	43.53
lnc5150	8.77E-03	108.17	up	5.62E-03	23.42	up	RP11-348F1.3	GENCODE	717.43	650.11	20.76
lncagf.1	1.67E-03	24.02	up	6.11E-03	9.52	up	AK094692	UCSC	2320.13	3699.10	174.09
lnc9603	1.61E-04	2.02	up	7.59E-04	2.67	down	RP11-443C10.1	GENCODE	1001.64	632.53	691.01

[a] FC: fold change; LN: lupus nephritis; HC: healthy control

Table S3 Associations of the expression of plasma lnc7074 with categorical clinical parameters of systemic lupus erythematosus (SLE) patients

Characteristics	Number	lnc7074	*Z*	*P*
Active SLE patients				
Yes	144	0.67 (0.31, 1.30)	-0.941	0.347
No	96	0.51 (0.31, 1.02)		
Prednisone dose				
≥15 mg/day	124	0.70 (0.37, 1.42)	-1.827	0.068
<15 mg/day	116	0.51 (0.29, 1.02)		
Immunosuppressant				
Yes	187	0.60 (0.31, 1.14)	-0.293	0.770
No	53	0.51 (0.28, 1.76)		

Table S4 Associations of the expression of plasma linc0597 with categorical clinical parameters of systemic lupus erythematosus (SLE) patients

Characteristics	Number	linc0597	*Z*	*P*
Active SLE patients				
Yes	144	1.18 (0.73, 2.06)	-0.569	0.569
No	96	1.30 (0.79, 1.99)		
Prednisone dose				
≥15 mg/day	124	1.29 (0.65, 2.00)	-0.043	0.966
<15 mg/day	116	1.21 (0.79, 2.03)		
Immunosuppressant				
Yes	187	1.22 (0.75, 1.88)	-0.626	0.531
No	53	1.48 (0.75, 2.11)		

Table S5 Associations of the expression of plasma lnc0640 with categorical clinical parameters of systemic lupus erythematosus (SLE) patients

Characteristics	Number	lnc0640	*Z*	*P*
Active SLE patients				
Yes	144	1.37 (0.53, 2.58)	-0.733	0.464
No	96	0.99 (0.58, 1.99)		
Prednisone dose				
≥15 mg/day	124	1.45 (0.58, 2.53)	-1.609	0.108
<15 mg/day	116	0.96 (0.50, 2.19)		
Immunosuppressant				
Yes	187	1.15 (0.54, 2.26)	-0.936	0.349
No	53	1.43 (0.59, 2.68)		

Table S6 Associations of the expression of plasma lnc05150 with categorical clinical parameters of systemic lupus erythematosus (SLE) patients

Characteristics	Number	lnc5150	*Z*	*P*
Active SLE patients				
Yes	144	1.14 (0.63, 2.46)	-0.585	0.559
No	96	1.35 (0.77, 2.37)		
Prednisone dose				
≥15 mg/day	124	1.30 (0.66, 2.55)	-0.595	0.552
<15 mg/day	116	1.18 (0.70, 2.32)		
Immunosuppressant				
Yes	187	1.27 (0.72, 2.34)	-0.033	0.974
No	53	1.30 (0.55, 2.81)		

Table S7 Associations of the expression of plasma GAS5 with categorical clinical parameters of systemic lupus erythematosus (SLE) patients

Characteristics	Number	GAS5	*Z*	*P*
Active SLE patients				
Yes	144	0.45 (0.32, 0.76)	-0.471	0.638
No	96	0.49 (0.35, 0.78)		
Prednisone dose				
≥15 mg/day	124	0.47 (0.31, 0.80)	-0.222	0.824
<15 mg/day	116	0.48 (0.33, 0.71)		
Immunosuppressant				
Yes	187	0.48 (0.34, 0.74)	-0.440	0.660
No	53	0.47 (0.30, 0.82)		
